# Chloroform

**Published:** 1847-12

**Authors:** 


### CHLOROFORM.

Several operations have been performed, by Prof. Brainard, with very satisfactory results, upon patients while under the influence of this new agent for producing insensibility to pain, which Prof. Blaney manufactured in the College Laboratory. E.

